# Mifepristone Treatment in Four Cases of Primary Bilateral Macronodular Adrenal Hyperplasia (BMAH)

**DOI:** 10.1210/jc.2018-02638

**Published:** 2019-05-21

**Authors:** Pejman Cohan, Honey E East, Sandi-Jo Galati, Jennifer U Mercado, Precious J Lim, Michele Lamerson, James J Smith, Anne L Peters, Kevin C J Yuen

**Affiliations:** 1 Specialized Endocrine Care Center, Beverly Hills, California; 2 Baptist Premier Medical Group, Jackson, Mississippi; 3 Endocrine and Diabetes Specialists of Connecticut, Trumbull, Connecticut; 4 Swedish Pituitary Center, Departments of Neuroendocrinology and Neurosurgery, Swedish Neuroscience Institute, Seattle, Washington; 5 Corcept Therapeutics, Menlo Park, California; 6 Keck School of Medicine, University of Southern California, Los Angeles, California; 7 Barrow Pituitary Center, Departments of Neuroendocrinology and Neurosurgery, Barrow Neurological Institute, University of Arizona College of Medicine, Phoenix, Arizona

## Abstract

**Context:**

Primary bilateral macronodular adrenal hyperplasia (BMAH) is a rare form of adrenal Cushing syndrome conventionally treated with adrenalectomy. Medical treatment is often reserved for patients not eligible for surgery. However, to date there have been few studies about the efficacy of mifepristone for the treatment of BMAH associated with hypercortisolism.

**Objective:**

To describe a series of patients with hypercortisolism due to BMAH treated with mifepristone from multiple medical practices.

**Design:**

We retrospectively assessed four patients treated with mifepristone for hypercortisolism due to BMAH who had either failed unilateral adrenalectomy, declined surgery, or were poor surgical candidates.

**Results:**

Mifepristone induced clinical improvement and remission of the signs and symptoms of hypercortisolism in all described patients with BMAH. The median treatment duration at the time of efficacy response assessment was 5 months (range: 3 to 18 months). Improvement in cardiometabolic parameters was observed as early as 2 weeks after treatment was started. All patients achieved improvements in glycemic control and hypertension and had significant weight loss. The most common adverse event observed with mifepristone therapy was fatigue. Increases in TSH level occurred in two patients.

**Conclusion:**

Mifepristone can be an effective medical alternative to surgery in patients with hypercortisolism due to BMAH.

## Teaser

Mifepristone treatment may be an effective alternative to bilateral adrenalectomy in patients with hypercortisolism due to primary BMAH.

Primary bilateral macronodular adrenal hyperplasia (BMAH) is a rare cause of adrenal Cushing syndrome characterized by nonpigmented nodules >1 cm in diameter (macronodules) (1, 2). The degree of hypercortisolism in BMAH is often mild and insidious, although rarely, serious forms can be identified (3). BMAH is reported predominantly in women; however, the true prevalence of BMAH in the general population is unknown (4, 5). The pathogenesis of BMAH involves aberrant or amplified hormone receptor expression and intra-adrenal secretion of corticotropin (2, 4, 6, 7). Actors of the cAMP/protein kinase A signaling pathway or genes causing a hereditary familial tumor syndrome, including adenomatous polyposis coli gene (*APC*), menin (*MEN1*), and fumarate hydratase (*FH*), favor the development of BMAH (5). Additionally, in recent years the identification of a mutation of the tumor suppressor gene, armadillo repeat containing 5 (ARMC5), via combined pan-genomic approaches has also been implicated as a frequent cause of apparently sporadic or familial BMAH (3). Somatic second-hit mutations of ARMC5 have been found in individual adrenal nodules of patients carrying germline ARMC5 mutations (8). When feasible, genetic screening should be considered because it offers a good opportunity for early diagnosis by familial screening, treatment planning, and administration of early therapy.

Up to 15% of adrenal adenomas are bilateral (9, 10), with less severe cortisol hypersecretion being more common in patients with bilateral adrenal adenomas compared with their unilateral counterparts (41.5% vs 12.2%) (11). Patients with BMAH may not present with overt signs and symptoms of Cushing syndrome (*e.g.,* facial plethora, proximal muscle weakness, moon facies). However, even so-called mild hypercortisolism is associated with several comorbidities, such as type 2 diabetes mellitus (T2DM), hypertension, osteoporosis, dyslipidemia, and obesity (12–14), as well as increased cardiovascular risk and mortality (15–17).

Bilateral adrenalectomy (BLA) has conventionally been considered the treatment of choice for BMAH, but it leads to permanent adrenal insufficiency, necessitating lifelong glucocorticoid and mineralocorticoid replacement and reduced quality of life compared with the general population (18, 19). Unilateral adrenalectomy has recently been proposed as an alternative in select patients, with some advocating for the removal of the larger gland, especially in older patients (20). However, recurrent or persistent hypercortisolism after partial adrenalectomy has been previously reported (20–24) and may be attributed to the initial functionality or subsequent enlargement of the contralateral adrenal gland that continues to secrete cortisol. In these cases, another surgery to remove the remaining adrenal gland or lifelong medical therapy may be necessary. Studies examining recurrence of BMAH after partial adrenalectomy found divergent rates of recurrence: one study of 15 patients found a 13% recurrence rate in patients with at least 5 years of follow-up (21), and another of 12 patients demonstrated a 67% recurrence rate after 4.5 years of follow-up (24).

Mifepristone (Korlym^®^, Corcept Therapeutics, Menlo Park, CA) is a competitive glucocorticoid receptor antagonist medical therapy that is approved by the US Food and Drug Administration to treat hyperglycemia in patients with Cushing syndrome regardless of etiology. Because of the rarity of BMAH, published data on mifepristone and other medical therapies related to BMAH are limited (Table 1) (24–34). Herein, we describe a series of patients with hypercortisolism due to BMAH who were effectively treated with mifepristone.

**Table 1.** Recent Reports on the Use of Medical Therapy in BMAH

Reference	Number of Patients	Treatment	Duration	Outcome
Obata *et al.* 2011 (25)	1	Trilostane	7 y	Reduction of serum cortisol to upper limit of normal
				Improvement in moon facies and weight
				Massive enlargement of adrenal glands
Comte-Perret *et al.* 2014 (26)	1	Ketoconazole	10 y	Reduction of UFC to ULN
				Minimal increase in adrenal glands
				Recovery of ACTH
				Persistent suppressed DHEA-S
Hannah-Shmouni *et al.* 2018 (27)	1	Leuprolide acetate	10 y	Control of macromastia and mastodynia
Karapanou *et al.* 2013 (28)	1	Leuprolide acetate	3.3 y	Biochemical control
				Recovery of ACTH
Albiger *et al.* 2015 (24)	2	Propranolol	Not reported	Reduction in 24-h UFC
				Persistent suppressed ACTH
				Worsening of glycemic control and weight gain
Oki *et al.* 2009 (29)	1	Propranolol	2 y	Reduction in cortisol secretion
				No improvement in glucose metabolism
Mazzuco *et al.* 2009 (30)	1	Propranolol	8 mo	Reduction in cortisol secretion
				Improvement in Cushingoid features
Albiger *et al.* 2015 (24)	1	Octreotide LAR	5 mo	Reduction in UFC
				Modest clinical improvement
				Patient eventually underwent surgery
Karapanou *et al.* 2013 (28)	1	Octreotide LAR	3 mo	No control
Preumont *et al.* 2011 (31)	1	Octreotide	6 mo	No control
		Pasireotide	3 mo	No control
Castinetti *et al.* 2009 (32)	1	Mifepristone	6 mo	Improvement in hypertension and Cushingoid features
				Discontinuation of metformin after 1 mo; decrease in HbA1c from 7.1% to 6.4% after 6 mo
Moraitis and Auchus 2015 (33)	1	Mifepristone	3 mo	Improvement in weight, Cushingoid features, insomnia, muscle weakness, and libido
				Recovery of ACTH and DHEA-S
Bourdeau *et al.* 2016 (34)	9	*β*-Blockers: propranolol, nadolol, atenolol, pindolol	Up to 15 y^*a*^	Reduction in cortisol response to posture test reported in 7 patients
				Partial reduction in UFC in patient with overt hypercortisolism. Patient underwent unilateral adrenalectomy followed by continued *β*-blocker therapy, which normalized UFC.
				Reduction in UFC but no reduction in cortisol response to posture test. UFC increased over time, and patient developed signs of Cushing syndrome. Patient underwent BLA.

^a^ Treatment duration not always given.

## Methods

A series of four patients with hypercortisolism secondary to BMAH is described in detail to highlight the diagnostic challenges and to illustrate a safe and effective medical treatment strategy. These cases reflect the clinical observations reported from four individual clinical practices across the United States. Institutional review board approval was not required, and informed consent was obtained from each patient.

## Results

Case series results are summarized in Tables 2 and 3 and are individually described here.

**Table 2.** Summary of Characteristics and Laboratory Findings at Presentation

	Case 1	Case 2	Case 3	Case 4
Age at diagnosis, y	71	54	71	27
Sex	F	F	M	F
BMI, kg/m^2^ (normal <30 kg/m^2^)	19.5	43	33.2	34.3
Fasting plasma glucose, mg/dL (normal 65–99 mg/dL)	253	139	116	115
HbA1c, % (<5.7%)	7.9	8.5	6.1	6.3
Blood pressure, mm Hg (normal 120/80 mm Hg)	129/70	141/88	170/90	156/104
Symptoms	Bruising	Cushingoid features	Headaches	Cushingoid features
	Hematuria	Extreme weakness	Anxiety	Headaches
			Insomnia	Anxiety
			Weight gain	Insomnia
				Depression
				Poor memory and concentration
				Dizziness
				Precipitous weight gain
Cortisol after 1-mg DST, μg/dL (normal <1.8 μg/dL)	18	18.8	2.6	—
LNSC, ng/mL^*a*^	5.075	0.6	0.62	
	2.43	3.9	1.23	4.06
	(ULN <1.56 ng/mL)	2.0(ULN <1.5 ng/mL)	(ULN <1.56 ng/mL)	4.284.935.45.58
				5.62
				10.26
				(ULN <1.49 ng/mL)
UFC, μg/d (normal 4–50 μg/d)	116	18.2	34.5	444.2
	32	21.3		292.3
	69			231.7
	74			
	57			
	77			
ACTH, pg/mL (normal 6–50 pg/mL)	13	Undetectable	Undetectable	Undetectable
	Undetectable^*b*^			
	8.1^*b*^			
	14.6^*b*^			
DHEA-S, μg/dL	82 (reference range 7–177 μg/dL)	36 (reference range 8–188 μg/dL)	—	—
Size of nodules, cm	1.7 (left), enlargement (right)	Diffuse enlargements	1.9–3.5	1.5–2.9

To convert cortisol and LNSC to nmol/L, multiply by 27.6.

To convert UFC to nmol/d, multiply by 2.76.

To convert ACTH to pmol/L, multiply by 0.22.

To convert DHEA-S to µmol/L, multiply by 0.0271.

^a^ LNSC values are from different laboratories with different assays and reference ranges.

^b^ Values from tertiary center.

**Table 3.** Assessments Before and After Mifepristone Treatment

	Case 1	Case 2	Case 3	Case 4	
Duration of mifepristone treatment at data collection	4 mo	18 mo	3 mo	2 wk	6 mo
Maximum dosage	300 mg 3 times/wk	600 mg once daily	300 mg once daily	600 mg once daily	900 mg once daily

	Before	After	Before	After	Before	After	Before	After	
ACTH, pg/mL (normal 6–50 pg/mL)	<5	108	<5	54	<5	29	<1.1	<1.1	<5
Fasting plasma glucose, mg/dL (normal 65–99 mg/dL)	253	121	139	113	116	99	115	91	85
HbA1c, % (normal <5.7%)	8.1	6.7	8.5	5.7	6.1	5.8	6.3	—	—
Blood pressure, mm Hg (normal 120/80 mm Hg)	129/70	127/81	141/88	137/81	170/90	140/80 Patient has PA treated with spironolactone	156/104	122/94	98/62
Weight loss, kg	0		23.2		2.3			10.9	10
Side effects/intervention	Fatigue/none		Gastrointestinal discomfort/reduction in mifepristone dosage		Heat intolerance/none		Fatigue, nausea, headache/none		
	TSH increase/levothyroxine						TSH increase/levothyroxine		
			Fatigue/none						
			Hypokalemia/spironolactone dosage increase						
				
Clinical outcomes	Reduction in HbA1c despite complete liberalization of diet		Resolution of diabetes with discontinuation of insulin		Reduction in blood pressure and HbA1c		Resolution of blood pressure and glucose parameters		
			Decreased blood pressure with less medication		Improved anxiety, energy, sleep		Palliation of Cushingoid features		
							Improved sleep, energy, cognitive function, and mood		

To convert ACTH to pmol/L, multiply by 0.22.

To convert fasting plasma glucose to mmol/L, multiply by 0.0555.

### Case 1: mifepristone after failed surgery

**Figure 1.**
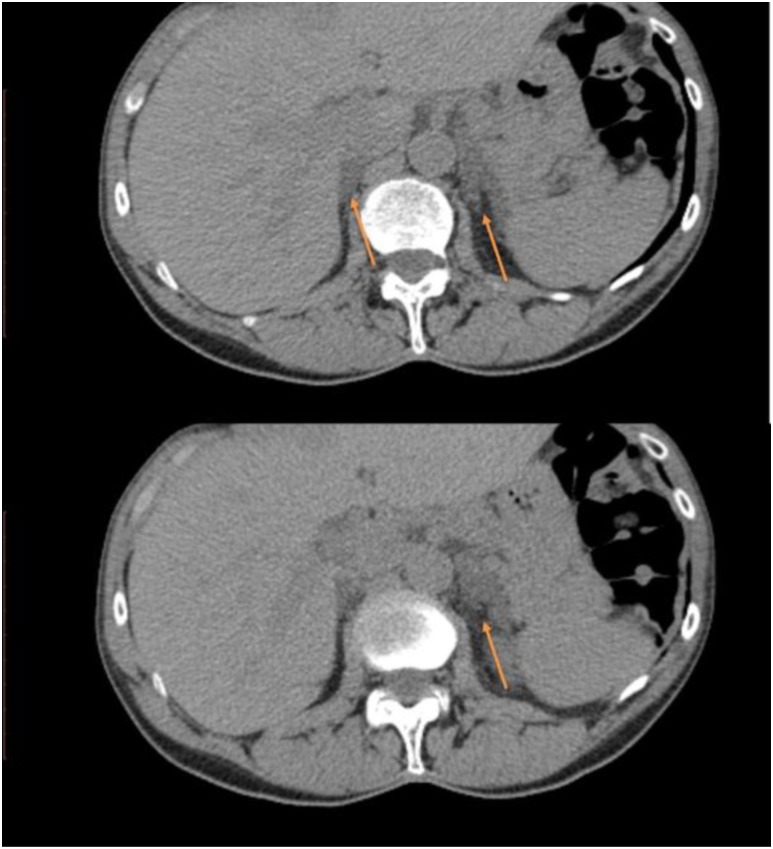
CT images from case 1 showing an enlarged right gland and nodular hyperplasia on the left gland (arrows).

A 71-year-old woman presented with hematuria and underwent an abdominal CT scan (Fig. 1), which revealed a 1.7-cm left adrenal nodule and an enlarged right adrenal gland. Her past medical history included nephrolithiasis at age 41, T2DM at age 58, treatment with metformin (HbA1c 7.9%, normal <5.7%), a long history of easy skin bruising that worsened at age 66, and osteoporosis with a T-score of −2.6 at the left femoral neck. Physical examination was notable only for bruising on her lower extremities.

Initial biochemical workup (Table 2) revealed 24-hour urine free cortisol (UFC) findings with levels ranging from 32 µg/d (88 nmol/d) to 116 µg/d (320 nmol/d) [upper limit of normal (ULN) <50 µg/d (<138 nmol/d)], elevated mean late-night salivary cortisol (LNSC), unsuppressed serum morning cortisol on overnight 1-mg dexamethasone suppression test (DST) with a normal dexamethasone level of 321 ng/dL (8179 pmol/L) [reference range 180 to 550 ng/dL (4586 to 14014 pmol/L)], and normal serum morning ACTH.

The patient opted for a second opinion at a tertiary center, where serial hypothalamic-pituitary-adrenal (HPA) testing revealed 24-hour UFC levels of 62.6 µg/d (173 nmol/d) and 50.9 µg/d (140 nmol/d) [ULN <45 µg/d (<124 nmol/d)], LNSC levels of 0.83 ng/mL (2.29 nmol/L) and 1.9 ng/mL (5.24 nmol/L) [ULN <1.0 ng/mL (2.76 nmol/L)], and a late-night serum cortisol of 70 ng/mL (193 nmol/L [ULN <75 ng/mL (<207 nmol/L)]. A dexamethasone-CRH (Dex-CRH) test found a cortisol level of 1.8 μg/dL (50 nmol/L) [ULN <1.8 μg/dL (<50 nmol/L)] at 0 minutes, followed by a cortisol level of 2 μg/dL (55 nmol/L) [ULN <1.4 μg/dL (<39 nmol/L)] at 15 minutes. Serial morning ACTH levels ranged from <5 to 14.6 pg/mL (<1.1 to 3.2 pmol/L) [normal range 6 to 50 pg/mL (1.3 to 11 pmol/L)], and serial evening ACTH levels ranged from 6.9 to 9.9 pg/mL (1.5 to 2.2 pmol/L) [normal <20 pg/mL (<4.4 pmol/L)]. Because the nodule was small and biochemical tests were equivocal, the treating physician did not recommend surgery. Instead, mifepristone was initiated at 300 mg once daily. After 1 week of treatment, the patient reported fatigue, dizziness, and leg swelling. She developed mild hypokalemia, with a potassium level of 3.4 mEq/L (normal range 3.5 to 5.0 mEq/L).

Although advised of these expected adverse events, she elected to stop mifepristone and undergo left laparoscopic adrenalectomy. She subsequently experienced fatigue and dizziness postoperatively and was promptly placed on hydrocortisone replacement therapy. Pathology revealed BMAH with a predominant nodule.

One month postoperatively, hydrocortisone was held for 36 hours, and assessment of serum cortisol showed elevated morning serum cortisol of 25 μg/dL (690 nmol/L) [normal range 4 to 22 µg/dL (110 to 607 nmol/L)]. Because of this result, hydrocortisone was discontinued. At postoperative month 3, her 24-hour UFC was 71.2 μg/d (197 nmol/d) [normal <50 μg/d (138 nmol/d)], and LNSC was 1.56 and 0.32 ng/mL (4.3 and 0.9 nmol/L) [ULN <1.56 ng/mL (<4.3 nmol/L)]. At follow-up, abdominal CT revealed postoperative changes in the left adrenal bed with surgical clips. The right adrenal was unchanged. At 6 months, her 24-hour UFC was 78.4 μg/d (216 nmol/d), and LNSC was 3.26 and 2.54 ng/mL (9.0 and 7.0 nmol/L), suggesting persistence of hypercortisolism. During this time, there was no improvement in her primary symptoms. Excessive bruising continued, and her HbA1c worsened to 8.1%.

At this point, mifepristone was restarted. Because of her previous objection to daily dosing, a dosage of 300 mg once a week was initiated, which was gradually increased to 300 mg three times a week. Three months later, her HbA1c decreased to 7.4%. She initially reported fatigue, which resolved without intervention. She also developed mild increases in serum TSH levels, whereas serum free T4 and free T3 levels were normal, and antithyroid antibodies were negative. She started levothyroxine, and her neurocognitive impairment and depression resolved. Her HbA1c further decreased to 6.7% after 4 months of mifepristone therapy despite complete liberalization of her diet (Table 3). Her HbA1c was maintained until 8 months after restarting therapy, when it increased to 7.3%. Concurrently, she also experienced recurrence of cognitive impairment despite taking thyroid hormone and normalized TSH. Positron emission tomography CT of the brain disclosed moderate cortical atrophy, marked decreased activity in the parietal cortex bilaterally, and moderately decreased activity in the temporal cortex bilaterally, consistent with a neurodegenerative process, probably Alzheimer’s disease. Fourteen months after restarting mifepristone, the patient was admitted to the hospital for altered mental status. At the time of hospital admission, the patient was hypertensive (blood pressure 165/85 mm Hg) and hyperglycemic [glucose 177 mg/dL (9.8 mmol/L)], making adrenal insufficiency unlikely. Regardless, mifepristone was immediately stopped, and the patient subsequently died of a cardiovascular event prompted by a subcentimeter subacute right frontal lobe infarct found on MRI.

### Case 2: mifepristone in a patient who declined surgery

A 54-year-old woman presented with Cushingoid features and extreme muscle weakness necessitating the use of a cane to ambulate. Her medical history included uncontrolled T2DM (HbA1c 8.5% on short-acting sliding scale insulin and insulin glargine 20 U at bedtime), resistant hypertension (blood pressure 141/88 mm Hg) despite administration of six antihypertensive agents, obesity [body mass index (BMI) 43 kg/m^2^], dyslipidemia, congestive heart failure, chronic obstructive pulmonary disease, sleep apnea, chronic renal insufficiency, and hyperthyroidism. Her blood pressure medications included amlodipine 10 mg once daily, carvedilol 25 mg twice daily, furosemide 40 mg once daily, hydralazine 50 mg three times daily, lisinopril 5 mg once daily, and clonidine 0.2 mg once daily.

**Figure 2.**
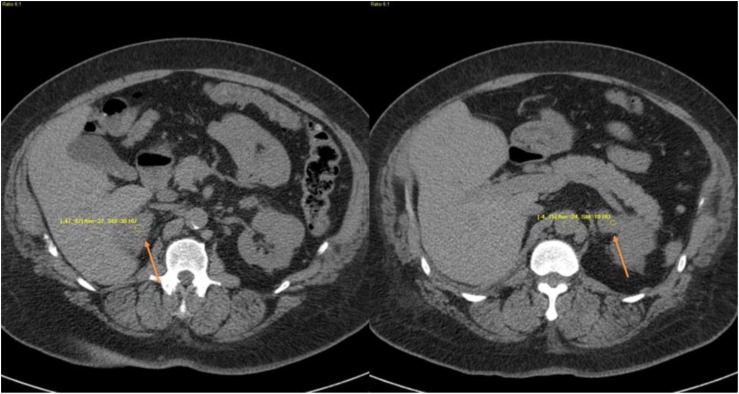
CT images from case 2 showing diffuse bilateral enlargements (arrows).

The patient’s Cushingoid features and clinical presentation (uncontrolled T2DM and resistant hypertension) prompted screening for hypercortisolism. Biochemical testing indicated ACTH-independent hypercortisolism: multiple ACTH measurements <5 pg/mL (<1.1 pmol/L), unsuppressed cortisol after 1-mg DST, mean 24-hour UFC 19.8 µg/d (54.6 nmol/d) [normal <50 µg/d (<138 nmol/d)], mean LNSC 2.1 ng/mL (5.79 nmol/L) [normal range 1.0 to 1.5 ng/mL (2.76 to 4.14 nmol/L)], and low dehydroepiandrosterone sulfate (DHEA-S) (Table 2). Additional biochemical screening ruled out primary aldosteronism (PA) and pheochromocytoma. Abdominal CT scans revealed diffuse bilateral enlargement of the adrenal glands, indicating BMAH (Fig. 2).

The patient declined BLA, and mifepristone was subsequently initiated at 300 mg once daily. After 2 weeks, her fasting plasma glucose was 128 mg/dL (7.11 mmol/L), her blood pressure decreased to 118/80 mm Hg, and the patient lost 1.4 kg. Mifepristone dosage was titrated to 600 mg once daily, and after 6 months of treatment her HbA1c was 5.8%, and she lost 5 kg. After 12 months of mifepristone treatment, her HbA1c had decreased to 5.6%, and she discontinued all insulin therapy. Her weight had decreased a total of 12.7 kg, and her blood pressure had decreased to 130/84 mm Hg. After 18 months of mifepristone treatment, HbA1c without insulin was stable at 5.7%, her weight had decreased by 23.2 kg, she no longer needed a cane for ambulation, and her blood pressure was controlled (137/81 mm Hg) with fewer medications (amlodipine 5 mg once daily, lisinopril 5 mg once daily, clonidine 0.2 mg once daily) (Table 3). Over the course of mifepristone treatment, she developed hypokalemia, and spironolactone was initiated and titrated up to 100 mg twice daily, based on potassium and plasma renin activity. The patient continues to use mifepristone daily.

### Case 3: mifepristone in a patient with primary aldosteronism and cortisol cosecretion who declined surgery

A 71-year-old man with resistant hypertension was referred for suspected PA. He presented with daily debilitating headaches and was found to have severe hypertension (blood pressure of 230/100 mm Hg). Despite initiation of five antihypertensive agents, his blood pressure remained elevated (170/90 mm Hg). Previously performed plasma metanephrines were normal, as were the patient’s 24-hour UFC [34.5 µg/d (95.2 nmol/d); normal <50 µg/d (<138 nmol/d)] and renal artery Doppler examination. His physical examination was notable only for central obesity. He weighed 108.4 kg, and his BMI was 33.2 kg/m^2^. Further laboratory testing revealed an aldosterone/renin ratio of 53 ng/dL:ng/mL/h, with serum potassium of 3.8 mEq/L (normal range 3.5 to 5.0 mEq/L), suggesting PA.

**Figure 3.**
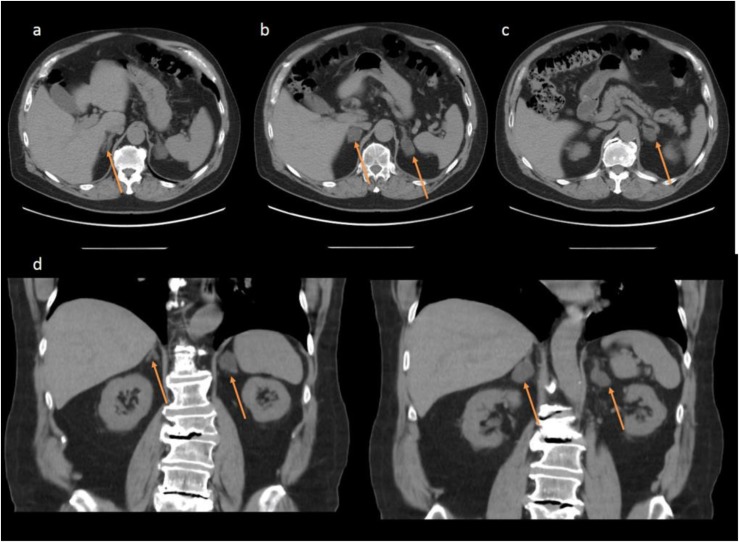
CT images from case 3 demonstrating bilateral adrenal nodules. (a–c) Multiple axial views of adrenal nodules, indicated by arrows. (d) Coronal images of bilateral adrenal nodules (arrows).

An oral salt load test confirmed PA with a 24-hour urine aldosterone of 17.9 µg/d (49.6 nmol/d). An abdominal CT scan revealed four bilateral adrenal adenomas ranging from 1.9 to 3.5 cm in diameter (Fig. 3). The patient declined adrenal vein sampling and surgery. Biochemical testing for cortisol revealed random ACTH level <5 pg/mL (<1.1 pmol/L), unsuppressed cortisol after 1-mg DST, and normal LNSC (Table 2). BMAH was diagnosed based on imaging and biochemical evaluation.

Spironolactone was initiated for PA at 25 mg/d and titrated to 100 mg/d over 3 months, with subsequent reduction in concomitant medications. He remained on valsartan/hydrochlorothiazide 320/25 mg once daily, with a resulting blood pressure of 140/80 mm Hg.

The patient developed mastodynia associated with spironolactone use. A trial of eplerenone (50 mg) was initiated, but the patient’s hypertension worsened, and spironolactone was resumed.

Over the next year, the patient developed worsening anxiety, insomnia necessitating pharmacologic treatment, weight gain of 4.5 kg, and prediabetes with HbA1c 6.1% and fasting plasma glucose 116 mg/dL (6.4 mmol/L) [normal range 65 to 99 mg/dL (3.6 to 5.5 mmol/L)]. Repeat HPA axis tests showed further elevated cortisol after 1-mg DST of 3.4 μg/dL (93.8 nmol/L) [ULN <1.8 μg/dL (<50 nmol/L)] and normal LNSC of 0.83 nmol/L (ULN <4.3 nmol/L).

Subsequently, the physician and patient decided to start mifepristone at a dosage of 300 mg/d. After 3 months of mifepristone treatment, HbA1c and fasting plasma glucose decreased to 5.8% and 99 mg/dL (5.5 mmol/L), respectively. He lost 2.3 kg and reported improvements in sleep, energy, and anxiety. ACTH rose to 29 pg/mL (6.4 pmol/L), suggesting recovery of the HPA axis.

### Case 4: presurgical use of mifepristone

A 27-year-old woman who desired pregnancy presented for evaluation of possible hypercortisolism. She was previously healthy until ∼3 years before presentation, when she began experiencing headaches, menstrual irregularities, and unexplained weight gain. At presentation, she was found to have new-onset hypertension (blood pressure of 156/104 mm Hg), prediabetes [HbA1c 6.3% and fasting plasma glucose 115 mg/dL (6.4 mmol/L)], precipitous weight gain of 30 kg in 2 years (BMI at presentation 34.28 kg/m^2^), and secondary amenorrhea.

The patient complained of insomnia, depression, anxiety, weakness, nausea, dizziness, and poor memory and concentration. She displayed overt Cushingoid features with dorsocervical and supraclavicular fat pads, abdominal striae, excessive body and facial hair, skin bruises, acne, and facial plethora.

**Figure 4.**
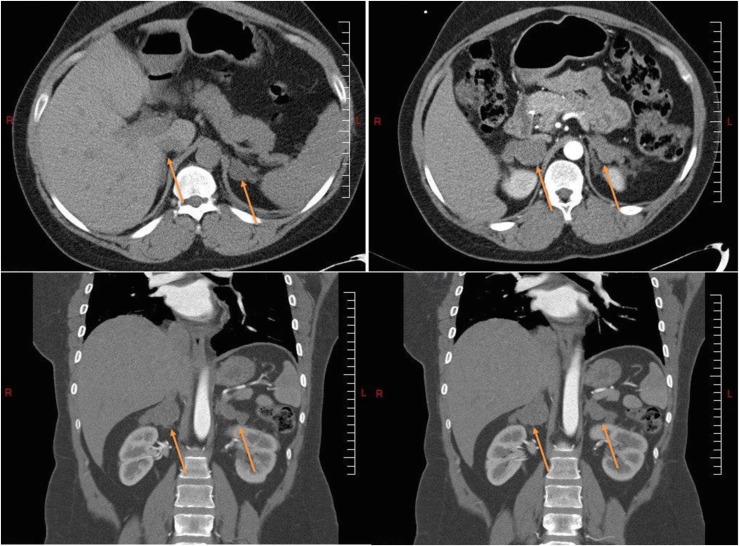
CT images from case 4 showing sagittal and coronal views of bilateral macronodular hyperplasia (arrows).

Biochemical testing indicated severe hypercortisolism: mean 24-hour UFC 322.7 µg/d (891 nmol/d) [ULN <50 µg/d (<138 nmol/d)], mean LNSC 5.73 ng/mL (15.8 nmol/L) [ULN <1.49 ng/mL (<4.1 nmol/L)], and mean morning ACTH <1.1 pg/mL (0.24 pmol/L) [normal range 7.2 to 63.3 pg/mL (1.6 to 13.9 pmol/L)]. Primary aldosteronism and pheochromocytoma were ruled out. Abdominal CT revealed symmetrically enlarged adrenal glands with nodules measuring 1.5 to 2.9 cm (Fig. 4).

Severe hypercortisolism placed her at risk for thromboembolic events and opportunistic infections, making her ineligible for immediate BLA. Medical therapy was administered to improve her underlying condition in preparation for BLA. Because a rapid response was desired, mifepristone was initiated at 300 mg/d. After 2 weeks, her blood pressure decreased to 122/94 mm Hg, her fasting plasma glucose decreased to 91 mg/dL (5.1 mmol/L), and her BMI decreased to 32.43 kg/m^2^.

Mifepristone was titrated to 900 mg/d over 6 months, with resolution of all comorbidities. Her hypertension resolved, fasting plasma glucose levels decreased, and BMI further decreased to 25.26 kg/m^2^ (Table 3). While on mifepristone, she experienced an increase of serum TSH levels, which resolved with levothyroxine, and cortisol withdrawal symptoms, which were transient and resolved without intervention. Improvement in signs and symptoms of hypercortisolism were noted, including weight loss of 20.9 kg, lightening of striae, reduction of supraclavicular and dorsocervical fat, decreased frequency of headaches, and improved sleep, energy, cognitive function, and mood.

A BLA was performed without complication 1 week after the patient discontinued mifepristone. She was treated with low-dose hydrocortisone replacement therapy and fludrocortisone (which she continues at maintenance dosages). The patient reported no symptoms of post-BLA adrenal insufficiency. Within 1 month of adrenalectomy, her menstrual periods returned spontaneously. Nine months after BLA, she still had not experienced symptoms of adrenal insufficiency, her ACTH levels were detectable, and she was able to conceive.

## Discussion

This case series suggests that medical therapy with mifepristone to treat hypercortisolism associated with BMAH is an effective and safe alternative or adjunctive treatment modality to bilateral or unilateral adrenalectomy. BLA leads to permanent adrenal insufficiency and the need for lifelong glucocorticoid and mineralocorticoid replacement. Patients with adrenal insufficiency are at high risk for potentially fatal adrenal crises (35). A pooled analysis of 162 patients found persistent adrenal tissue in 23% of patients who have had a BLA (36). Treatment with unilateral adrenalectomy can result in recurrence of hypercortisolism with rates as high as 67% because of enlargement of the contralateral gland (20–24). For some patients, surgery may be high risk (especially for some older adults), whereas other patients may decline surgery for any number of reasons, regardless of associated risk. Moreover, irrespective of the type of surgery, comorbidities stemming from hypercortisolism persist, leading to a poorer quality of life compared with the healthy population (37–39).

The literature regarding medical therapy for BMAH is limited (Table 1). Studies of trilostane, ketoconazole, leuprolide acetate, propranolol, and octreotide LAR reveal a mixed picture, often resulting in no improvement or even worsening of clinical outcomes such as glycemic control and weight gain. Two previous reports (n = 2 patients total) on mifepristone for the treatment of BMAH each demonstrated improvement in Cushingoid features, hypertension, or weight (32, 33). In our case series, each of the four patients, despite their varying circumstances, had clinical improvement of hypercortisolism with mifepristone.

Persistent hypercortisolism after failed unilateral adrenalectomy prompted the use of mifepristone in one patient (case 1). In this case, additional surgery to remove the contralateral gland was not a desired outcome, especially because the patient was elderly. Mifepristone was able to provide an alternative to BLA and resulted in clinical improvements that surpassed the results of previous unilateral adrenalectomies.

The patient preference to avoid adrenal surgery is another important consideration for medical therapy, as seen in cases 2 and 3. In such patients, some have previously advocated for active surveillance and management of comorbid conditions without addressing the underlying hypercortisolism. More recent studies show that these patients can develop new comorbidities or that their cardiometabolic profiles could worsen (39, 40). The development of postoperative adrenal insufficiency in 248 patients who underwent adrenalectomy for mild autonomous cortisol secretion (41) is additional evidence of the clinical relevance of hypercortisolism. In cases 2 and 3, managing comorbidities with antidiabetes medications and antihypertensives did not improve their cardiometabolic profiles. Their worsening metabolic comorbidities necessitated intervention with mifepristone, which resulted in immediate clinical benefit over that generated with their previous medications. In case 2, clinical benefits were seen in as little as 2 weeks, with improvements in hyperglycemia, hypertension, and body weight.

Because of its antiprogesterone activity, mifepristone cannot be used as lifelong treatment in a young woman desiring pregnancy, as in case 4. However, her hypercortisolism was so clinically and biochemically severe that a BLA could not be immediately performed. Because hypercortisolism causes a hypercoagulable state that is reflected by a >10-fold increased risk of venous thromboembolism (42), she was considered at high risk for surgery. In fact, the Endocrine Society and others have advocated for thromboprophylaxis in these types of patients (43, 44). Even postoperatively, the risk of thrombosis remains high (45). In this case, mifepristone was used to quickly ameliorate the severe hypercortisolism to prepare the patient for surgery. Mifepristone resulted in clinical benefit as early as the first 2 weeks of treatment, with improvements in hyperglycemia, hypertension, and weight. A dosage of 900 mg once daily over 6 months resulted in resolution of hyperglycemia, hypertension, and obesity, with palliation in all signs and symptoms of hypercortisolism. All these improvements lowered the patient’s operative risk and expedited recovery, as evidenced by the lack of postoperative adrenal insufficiency symptoms, spontaneous return of menses 1 month after surgery, and subsequent conception.

The most common adverse event observed with mifepristone therapy in our case series was fatigue, which resolved without intervention in all three patients who experienced it. Serum TSH level increases were observed in two patients, which necessitated initiation of levothyroxine. Reversible TSH increases in patients using mifepristone have been previously described in 19% of patients (46). The same report described hypokalemia in 44% of patients, resulting from overactivation of the mineralocorticoid receptor by cortisol. In our case series, hypokalemia was observed in only one patient, which resolved with an increase in the spironolactone dosage.

There are limitations to this case series, namely that the data are retrospective and were derived from multiple clinical practices. However, given the lack of available data, this case series of four patients offers a valuable addition to the literature on medical therapy for BMAH. Additional larger prospective studies are needed to determine whether long-term clinical improvements with mifepristone are sustainable and durable and whether the patient response to stress is altered. Published studies on short-term mifepristone treatment in normocortisolemic patients did not reveal any cases of adrenal insufficiency or crises (47). Another report of short-term mifepristone treatment in a patient with secondary adrenal insufficiency also did not result in adrenal crisis (48). However, it remains to be seen whether long-term treatment with mifepristone leads to different outcomes during intercurrent illnesses and stressors. Because ACTH and cortisol are expected to increase on mifepristone treatment, it also remains to be seen whether prolonged treatment has any effects on the proliferation and function of the remaining adrenal tissues.

In summary, our case series demonstrates the efficacy of mifepristone in treating patients with hypercortisolism associated with BMAH. Mifepristone elicited substantial clinical improvements and, in some cases, improvement in cardiometabolic parameters, with accompanying weight loss and improvement of Cushingoid features and neuropsychiatric symptoms after a median treatment duration of 5 months (range 3 to 18 months). It should also be noted that these improvements occurred concomitantly with a reduction or discontinuation of other medications. Mifepristone could be considered as a treatment option for patients with hypercortisolism associated with BMAH who decline surgery, experienced recurrence, or need to optimize conditions before BLA.

## Abbreviations:

ARMC5: armadillo repeat containing 5
BLA: bilateral adrenalectomy
BMAH: bilateral macronodular adrenal hyperplasia
BMI: body mass index
Dex-CRH: dexamethasone-CRH
DHEA-S: dehydroepiandrosterone sulfate
DST: dexamethasone suppression test
HPA: hypothalamic-pituitary-adrenal
LNSC: late-night salivary cortisol
PA: primary aldosteronism
T2DM: type 2 diabetes mellitus
UFC: urine free cortisol
ULN: upper limit of normal
